# Recovery of adults with autism spectrum disorder during intensive inpatient treatment: a qualitative study

**DOI:** 10.3389/fpsyt.2024.1383138

**Published:** 2024-06-07

**Authors:** Hendrikje Bloemert, Bram B. Sizoo, Elisabeth W. M. Verhoeven, Aartjan Beekman, Berno van Meijel

**Affiliations:** ^1^ Center for Developmental Disorders, Dimence Institute for Mental Health, Deventer, Netherlands; ^2^ Department of Nursing, Inholland University of Applied Sciences, Amsterdam, Netherlands; ^3^ Department of Psychiatry, Amsterdam Public Health Research Institute, Amsterdam Universitair Medische Centra (UMC; VUmc), Amsterdam, Netherlands; ^4^ Department of Clinical Psychology, University of Amsterdam, Amsterdam, Netherlands; ^5^ Leo Kannerhuis (Youz/Parnassiagroep), Nijmegen, Netherlands; ^6^ Parnassia Psychiatric Institute, Parnassia Academy, The Hague, Netherlands

**Keywords:** autism spectrum disorder, intensive inpatient treatment, recovery, adults, qualitative study

## Abstract

**Introduction:**

Although some adults with autism spectrum disorder (ASD) require intensive and specialized ASD treatment, there is little research on how these adults experience the recovery process. Recovery is defined as the significant improvement in general functioning compared to the situation prior to treatment.

**Methods:**

This qualitative study describes the recovery process from the perspective of adults on the autism spectrum during intensive inpatient treatment. Semi-structured interviews (n = 15) were carried out and analyzed according to the principles of grounded theory.

**Results:**

Our results indicate that, given the specific characteristics of autism, therapeutic interventions and goal-oriented work cannot be carried out successfully, and the recovery process cannot begin, if no good working relationship has been established, and if care is not organized in ways that a person on the autism spectrum finds clear and predictable.

## Introduction

In Dutch Mental Health Care (MHC) all referrals to mental health care must be made by a general practitioner (GP) either to basic mental health care (for common mental disorders), or to specialized mental health care (for severe mental disorders). Specialized MHC can be either in an outpatient or inpatient setting. The latter is reserved for very serious problems, involving, for example, acute suicidal risk of harm to others. These referral procedures also apply to people with Autism Spectrum Disorders (ASD). However, in case of persistent and severe psychiatric problems in patients with ASD that cannot be treated effectively in general mental health care, a referral can be made by a GP, psychologist or psychiatrist to a mental health care setting specialized in ASD However, the capacity of these specialized ASD facilities is very limited, especially regarding the inpatient services, with corresponding limited opportunities for placement and treatment. For a significant proportion of adults who experience problems associated with ASD, treatment in regular mental health care is sufficient. However, a smaller number requires more intensive and autism-specialized treatment, particularly those with severe symptoms and co-occurring conditions.

Comorbidity rates of 69 – 81% have been reported in adults on the autism spectrum (1, 2), with high prevalences of symptoms of depression, anxiety, attention-deficit/hyperactivity disorder (ADHD), psychotic disorder, substance use disorder (SUD), personality disorders, suicide and self-injury (1–9). Adults with severe autism symptoms and co-occurring conditions often require prolonged treatment over the life course, involving various treatment modalities in line with existing guidelines (10).

Research shows that adults on the autism spectrum, especially those with a combination of complex medical and mental health issues, often struggle to find services appropriate to their specific needs (11). It is also the case that limited specialized knowledge of autism in general mental health care can contribute to suboptimal treatment outcomes (12–16). In some cases, treatment may stagnate, leading to referral to other health care services without a sound rationale. This can also contribute to poor treatment outcomes, resulting in further deterioration of the person’s psychiatric condition, poorer psychosocial functioning, an increase in risky behavior (such as aggression, suicidal behavior and self-mutilation); and high healthcare utilization and costs (2, 9, 17). For such persons, the process of recovery is difficult and often prolonged.

Recovery is defined as the significant improvement in general functioning compared to the situation prior to treatment. Recovery is an interactive process of which the effects are experienced in different areas of life, involving distinctions between symptomatic, functional, social and/or personal recovery (18–23). Full symptomatic recovery is rare in people with serious mental conditions (24), and also in adults on the autism spectrum. However, when so-called personal recovery strategies are incorporated into a person’s treatment, long-term benefits can be obtained (22). Anthony (1993; p. 13) defined personal recovery as “a deeply personal, unique process of changing one’s attitudes, values, feelings, goals, skills and/or roles. It is a way of living a satisfying, hopeful, and contributing life even with limitations caused by the illness. Recovery involves the development of new meaning and purpose in one’s life as one grows beyond the catastrophic effects of mental illness” (25). Personal recovery may in turn contribute to symptomatic, functional, and social recovery (23).

Dutch mental health care offers specialized treatment for persons on the autism spectrum in both outpatient and inpatient settings. The rationale for these specialized setting is the assumption that persons with autism can benefit more from regular therapeutic interventions when they feel understood and accepted. Therefore these settings invest in a sound therapeutic milieu, and a trusting relationship between clients and staff prior to addressing comorbid conditions with state of the art interventions tailored to the needs of the individual client. Although we believe that this approach enhances recovery, to our knowledge, no systematic study to date has examined the recovery process of adults on the autism spectrum who receive intensive inpatient treatment in mental health care. Many questions therefore remain unanswered, such as which specific personal, treatment and contextual factors contribute towards a successful path to recovery.

For this reason, this qualitative study on the recovery of adults on the autism spectrum investigated personal process of recovery and the factors that influence it. The main research question was: How does the process of recovery of adults on the autism spectrum develop during intensive inpatient treatment, and which personal and environmental factors contribute to this process? The aim of the research was to gain more insight into the process of recovery of adults with ASD during intensive inpatient treatment. Based on these findings, we aimed for inductively developing a theoretical model of the process of recovery More knowledge in this area can contribute to the proper organization of personalized treatment, supporting the personal recovery process and providing opportunities to promote functioning and quality of life.

## Method

### Design

This sub-study was part of a larger research project exploring the factors that influence treatment intensity and recovery in people on the autism spectrum. To examine the process of recovery from a theoretical and interactional perspective, it followed a qualitative design using the grounded method approach. It was reviewed and approved by the Medical Ethics Review Committee at Amsterdam University Medical Center (UMC, location VUmc) (protocol no. 2017.120). It was also approved by the local scientific review board of the participating mental health institutions.

### Setting and participants

The study involved two institutions for mental health care in the Netherlands: Dimence Institute of Mental Health in Deventer and the Leo Kannerhuis Centre for Autism in Oosterbeek. Both institutions provide care for normal to high IQ patients. Participants were recruited, two months after discharge, from five departments with different intensities of treatment and autism-specialization: two ASD-specific High-Intensive Care (ASD-HIC) wards, and three general High-Intensive Care (general HIC) wards. The ASD-HIC wards provided autism-specific treatment, had selective admissions, and also a longer average length of stay than a general HIC ward. Treatment in ASD-HIC wards was based less on uniform protocols, but more on personalized treatment and on care that was in tailored to participants’ specific autism and co-occurring conditions, living situation and personal characteristics. On regular HIC-wards people are admitted who need acute psychiatric care for a wide range of psychiatric conditions, including ASD.

In order to study a general process of recovery in a variety of situations we included a broad population of adults on the autism spectrum treated in both ASD-specific and general HIC-settings. Additionally, we used a purposive sampling strategy that would include persons with different personal and contextual characteristics, such as age, gender, treatment setting and the status of admission (voluntary or involuntary). The inclusion and exclusion criteria are shown in **Table 1**.

**Table 1:** Inclusion and exclusion criteria for participation in the study.

Inclusion criteria: • a primary diagnosis of ASD according to DSM-5 criteria, and • age between 18 and 65 years, and • admission to an ASD High-Intensive Care (ASD-HIC) ward, or • admission to a general High-Intensive Care (general HIC) ward.
Exclusion criteria: • insufficient mastery of the Dutch language to participate in an interview, or • inability to communicate verbally.

After receiving information about this study, mental health practitioners at the participating departments invited persons who met the inclusion criteria to participate in the study. After expressing interest, 36 persons received written and oral information on the research project. Ultimately, 15 agreed to participate and gave informed consent. Ten participants were treated in an ASD-HIC and five in a general HIC.

### Data collection

Data was collected between July 2017 and January 2019. In the preparation phase, a focus-group interview was organized with three adults on the autism spectrum, three family members, and three healthcare providers. This interview aimed to explore the central theme of our study: the recovery process in adults on the autism spectrum, and the factors influencing it. Based on this exploration, we constructed a substantiated interview protocol with a topic list for the individual interviews. To determine whether the structure and language use was clear for participants in the study, a support-staff member with ASD reviewed the protocol and topic list.

The interviews were semi-structured with open-ended questions. All interviews were conducted during face-to-face conversations within two months of discharge. This was considered to be a timeframe within which participants would be able to recall their experiences during the process of recovery, including the factors that had contributed to their recovery.

According to the participant’s preference, the location of the interview was either the participant’s home or the treatment setting. The interviews were conducted by two researchers (HB and a research assistant) who had both been trained in qualitative interviewing techniques. In line with the principles of grounded theory, data collection and data analysis alternated to allow interim results to guide subsequent interviews. Data collection continued until sufficient data saturation had been achieved. After analysis of the twelfth interview, the model appeared to be sufficiently robust. The robustness of the model was confirmed during the analysis of the three subsequent interviews, where no new code words could be added to the code tree and no new information could be added to the existing code words. Based on this, the research team agreed that sufficient data saturation had been achieved after 15 interviews.

### Data analysis

The interviews were audio-recorded and transcribed verbatim. Their analysis followed the documented procedures of grounded theory (26). As well as the use of open and focused coding, and research memos, this included the constant comparison technique, an approach that allows concepts to emerge from the data rather than from placing the data in a preconceived framework.

The principal investigator (HB) was already familiar with the verbal data. To become even more familiar with it, she then read the transcripts line by line, subsequently analyzing the first three interviews with two authors (HB and BvM) and a research assistant. The remaining interviews were analyzed by the first author (HB), with continuous discussion and feedback sessions with the research team. MAXQDA Plus 12 ^©^ software was used for the analysis.

To identify data showing participants’ experiences of their process of recovery, *in vivo* codes were generated using the participants’ own wording. The codes were then merged into categories and sub-categories. By a process of constant comparison, the new cases were used to test the validity of these categories and subcategories, and also their inter-relationships. On this basis, the model was constructed with three main categories, namely (1) the situation before intensive inpatient treatment, (2) the conditions for achieving and sustaining recovery (i.e., the working alliance with care providers and organization of care) and (3) the process of recovery. The final model is constructed based on the codings that were categorized under these three main categories and the underlying codewords. The trustworthiness of this study was ensured in several ways. First, an interview protocol was developed before data collection began; this was carefully constructed on the basis of the literature and on the data from the focus-group session in which adults on the autism spectrum, family members, and mental health professionals participated. Second, interviews were conducted until data saturation was reached. Third, the research team met regularly to discuss and resolve discrepancies in (1) the identification, naming, and grouping of codes, subcategories, and categories; and in (2) the relationships between them. Finally, the researcher kept a logbook in which she described and justified the choices regarding the methodology and the actual execution of the study. These notes were discussed periodically within the research team.

## Results

Participants’ records were used to collect personal characteristics and background data (**Table 2**). Fifteen adults diagnosed with ASD participated in this study. They were aged 19 – 47 years (mean = 28.0, SD = 8.47). There were 10 women and five men. Ten participants had been treated in an ASD-HIC setting and five in a general HIC setting. Twelve of the 15 participants had one or more co-occurring conditions (**Table 2**).

**Table 2:** Sample characteristics (n=15).

	ASD-HIC	General HIC
**N**	10	5
Demographics		
Female	7 (70%)	3 (60%)
Mean age (range)	27 (20 – 47)	30 (19 – 47)
Number of co-occurring conditions		
No co-occurring condition	2 (20%)	1 (20%)
1 co-occurring condition	4 (40%)	2 (40%)
2 co-occurring conditions	3 (30%)	1 (20%)
3 co-occurring conditions	1 (10%)	1 (20%)
Type of co-occurring conditions*		
Attention deficit disorder	2	2
Anxiety disorder	1	–
Bulimia nervosa	1	–
Depression	2	2
Personality disorder not otherwise specified	1	–
Psychotic disorder not otherwise specified	–	1
Post-traumatic stress disorder	3	1
Somatoform disorder not otherwise specified	1	–
Substance-use disorders	2	1
Status of admission		
Involuntary	3 (30%)	4 (80%)

*based on diagnoses prior by admission.

**Figure 1:**
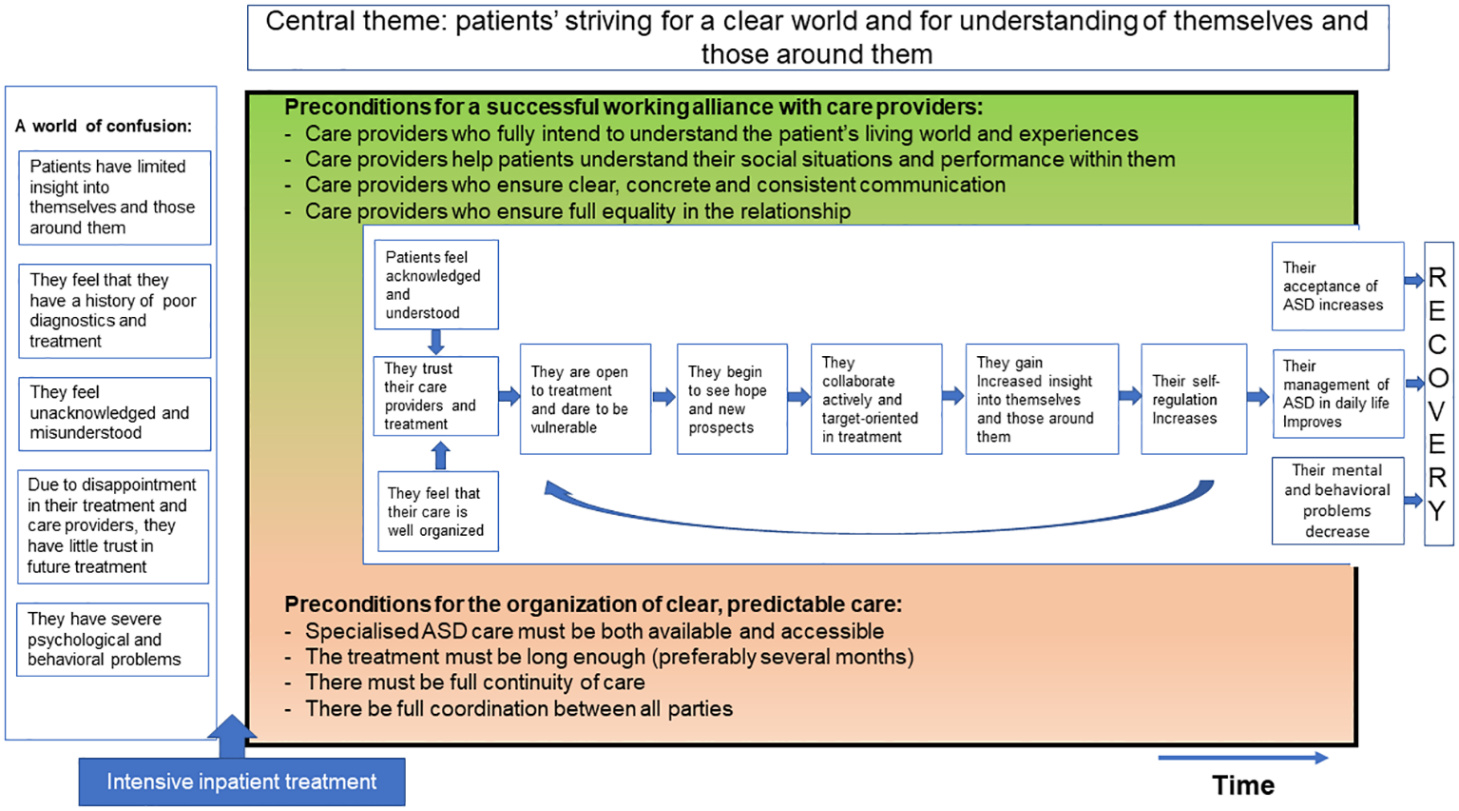
Model of recovery of patients with ASD during intensive inpatient treatment.

Based on the interview data, we inductively developed a model of recovery for adults on the autism spectrum after intensive inpatient treatment. The boxes on the left describe the participants’ situation before the start of intensive treatment. The green and pink fields specify the preconditions for a successful recovery process during intensive inpatient treatment, and the white field shows the recovery process that results if these preconditions are met. Below, we will elaborate further on this model (**Figure 1**).

### A world of confusion

Before the intensive inpatient treatment – and therefore before the start of the recovery process – many participants experienced the world as confusing. The following paragraphs explain the themes related to this confusing world. 

Most participants retrospectively reported having had limited insight into themselves and into the impact of autism on their functioning in daily life. In their experience, sensory overstimulation often led to a preoccupation with burdensome thoughts and feelings. As this increased stress and limited their ability to think clearly and act purposefully, they had experienced difficulties in carrying out meaningful daily activities.

Participants also reported that their information-processing was significantly different compared to other people. This gave them the feeling that they were not well understood by the outside world – which they, in turn, did not understand. They reported that challenging and often stressful interactions with people in their immediate environment reinforced their feelings of confusion and not being understood.

They lost control over their own functioning, which led them to avoid other people and withdraw from social life. They wanted to be independent of others, did not ask for help when they needed it, and persisted in their withdrawal even when things were not going well. Efforts to meet the perceived demands of the outside world often resulted in behavior they assumed to be socially appropriate.

*I have autism, of course. Your brain just works differently. It seemed as if the outside world didn’t understand me, and I didn’t understand the world around me. I just felt like an alien on this earth [female aged 25; ASD-HIC].*

Most participants reported that establishing the diagnosis of ASD had been significantly delayed because of their care providers’ limited expertise with autism. This contributed to the experience of receiving inadequate treatment. In the participants’ view, the treatment and care provided did not sufficiently meet their needs. Some also felt that the care was not well-enough organized, and that long waiting times made intensive and specialist treatment insufficiently accessible. Due to frequent changes of care provider, they often experienced a lack of treatment continuity.

*During a crisis admission, I was once told that I was not worth the insurance money. That was probably because the care provider simply didn’t know how to help me. When I panic, I can’t really communicate anymore, and when you’re in a clinic where they have absolutely no understanding of autism, they think that I’m just being uncooperative [female aged 25; ASD-HIC].*

Participants reported that the factors outlined above had a negative impact on their working alliance with care providers: they often felt that they were unacknowledged or misunderstood as human beings with specific problems and needs related to autism. This in turn further increased their feelings of confusion and loss of control over their lives.

*I didn’t feel that we were on the same page about how I was doing and what I needed. [The care provider] didn’t really understand me. I had the feeling that we were speaking a different language [female aged 27; ASD-HIC].*

In the participants’ view, inadequate treatment and poor organization of care caused them both to distrust and be disappointed in care providers and treatment programs. As this made them feel unsafe, they increasingly avoided contact with care providers and hesitated to share personal matters with them.

*I had the wrong diagnosis for a long time, and therefore had to fight very hard for myself in psychiatry. I found it very difficult to trust people and make contact with them. And because people sometimes overestimate me, they also think “she talks easily and can cope,” but I can’t [female aged 27; ASD-HIC].*

Due to their confusion, distrust, and lack of prospects, participants felt they were increasingly losing control over their lives. They felt like survivors, and many developed behavioral problems as a way of dealing with their problems and feelings of stress, such as aggression, suicidality, self-harm, alcohol abuse, drug abuse, or eating problems. Most participants also reported co-occurring conditions, such as depression, anxiety, psychosis, or trauma. Some reported that their psychiatric conditions restricted them from carrying out activities during the day, and that the resulting inactivity and social isolation often caused their psychiatric conditions to increase. In combination with psychiatric and behavioral problems, stressful living conditions led to admission to an HIC, sometimes with coercive measures. Some participants stated explicitly that they found these emergency admissions to be very traumatizing, as both treatment and care failed to meet their needs.

*So I’m sometimes taken literally off the railroad tracks, or I cut myself. Or I run away to the woods at night [when] my head is just so upset that it doesn’t think clearly [female aged 29; general HIC].*

### Conditions for achieving and sustaining recovery

Participants identified two important preconditions for a successful recovery process that led to a better understanding of themselves and those around them: (a) a good working alliance with care providers, and (b) clear and predictable organization of care.

a) Good working alliance with care providers

Participants stated that the quality of the working alliance had a great impact on their process of recovery. It was crucial for them to experience both that their care providers really wanted to understand them in their specific circumstances and context, and that they also understood the problems and challenges the participants encountered. Given that the participants often had difficulty putting their own problems and functioning into words, it was also important that their care providers took the time and made the effort to understand them as well as possible. They therefore appreciated it when care providers took a genuine interest in their experiences and lives by listening carefully and asking questions until they fully understood what the participants were saying and experiencing. This made participants feel heard and understood, and also increased their trust in care providers and the treatment program.

*They really looked at me as a person – by waiting, listening and asking questions. That allowed me to find my words [female aged 19; general HIC].*

Participants appreciated care providers who properly assessed and discussed their functioning and needs, and, when necessary, took a directive stance, responding proactively to problems and providing feedback. This gave them more insight into themselves, their functioning, and the influence of autism. In the end, it contributed to a better overview of the situation.

*They also saw all things I didn’t know about myself at all. That my voice changes when I’m tense, things like that. It was very special to me that they saw all of that [female aged 21; ASD-HIC].*

However, most participants on a general HIC ward who had barely experienced recovery, reported that their functioning was not well understood, and their needs had not been met properly by the care providers. The attitude of most care providers was non-directive. In these cases, a satisfying working alliance was not possible. Participants felt unsafe and did not trust the care provider. As a result, they withdrew from contact, and their psychological and behavioral problems increased.

*And then, when I’m in a panic, [the care provider] talks to me as if I’m misbehaving – tells me that I’m actually very clever, and should just act normal. “You’re an adult, so just use your intelligence,” he told me. That made me angry and sad – that’s how I get when I don’t feel understood [female aged 29; general HIC].*

Participants reported that, due to their difficulty in dealing with unpredictability and adequately overseeing situations, it was necessary for them that care providers helped to understand social and other situations and gave feedback on how they performed.

*Well, it all has to do with overstimulation. For example, when I went shopping with [the care provider], I came out of the shop and I was very happy. Then she asked me “how are you doing?” And I said “yes, I’m fine.” And then she said “let me see your hand,” and my hand went like this.... (hands shaking). You see, this is what I mean [female aged 25; ASD-HIC].*

Participants stated that feelings of unpredictability and the associated feelings of uncertainty were reduced by jointly drawing up a plan with concrete goals and steps for treatment, which allowed them and others to know what was going to happen. By creating clarity, insight into themselves, their autism, and their future prospects, this gave them more overall control over their lives.

Care providers’ communication style had an important impact on the participants’ recovery. Some participants stated that the process of recovery was limited if care providers failed to explain what they were going to do, or to keep appointments; or if they were unclear or unpredictable. This created confusion and loss of overview and control.

*But what I can’t stand is that people are erratic. Yes, that they react like this one time and like that the next. So you really don’t know what to expect of them [female aged 25; ASD-HIC].*

Due to their difficulties with information processing, it was very important to participants that care providers communicated clearly, concretely and consistently, and avoided ambiguous language.

*Sometimes it’s very nice when people just say things literally. It can be very helpful, and saves a lot of reading up. It prevents a lot of misunderstandings [female aged 19; general HIC].*

Participants reported that the experience of being valued by care providers was crucial to their recovery process. Important elements of this were equality in contact, being non-judgmental, cooperative and helpful, and offering participants enough room to make their own choices. If there was an equal and helping relationship, trust increased, and participants dared to show their vulnerability. This created a space in which they could discuss their treatment needs, which contributed to a higher acceptance of treatment and thus greater adherence. By leading to greater insight into themselves and their autism, and to greater acceptance of both, this gave them more control over their lives, which had a positive influence on reducing psychological and behavioral problems.

*It’s important that they don’t treat me like I’m someone who is very stubborn and very stupid. That they don’t treat you as if you don’t know anything yourself and that their way, the way they want you to do it, is the only right way. That doesn’t necessarily work for you. It works better when they say, “you can do it like this or like that,” but they leave you free to choose what you want and how you want to do it” [male aged 20; ASD-HIC].*

Some participants experienced an unequal relationship with caregivers who were judgmental and gave participants no room to make their own choices. As this made it impossible to be open and honest in their communication, it may have led to increased behavioral problems.

*Well, I must feel safe with people, of course, which also depends a lot on whether they will judge me or whether everything I say will remain confidential. And otherwise I tend to self-mutilate, or the eating goes wrong [female aged 19; general HIC].*

b) Organization of care:

Participants saw the organization of care as a second important precondition, as it had a major influence on their process of recovery.

Participants who had been in ASD-HIC reported that the availability and accessibility of specialized autism care was essential to recovery: it provided greater insight into their impairments and functioning, not only for people on the autism spectrum, but also for those around them. They also reported that this specialized treatment was essential to maintaining their improvement after clinical discharge, especially as people on the autism spectrum often find it difficult to generalize what they have learned into another context.

*I was admitted to the general HIC, though actually I was only there for my safety and not because I could be helped there or anything [female aged 29; general HIC].*

Participants from a general HIC ward did not receive specialized autism care; most barely experienced recovery. In their view, they did not receive appropriate treatment for their problems.

Participants from an ASD-HIC ward stated that treatment duration and planning had been very important to their recovery. On the one hand, they felt that they needed sufficient time to get used to the ward before they were able to participate fully in the treatment program. On the other hand, it was important for them to start therapies at an early stage in order to complete them as much as possible in the time available for inpatient treatment.

*It’s also very important to have enough time to work on yourself for the first time. It took me some time before I started to change. Nine months for treatment still isn’t very much, but it’s much more than in other settings [female aged 25; ASD-HIC].*

People on the autism spectrum found it important to have continuity of care providers. This created overview and clarity, and prevented anxiety and overstimulation. It were particularly participants from general HIC, who reported that confusion and distrust had been created by insufficient continuity of care, characterized by high numbers and changing of care providers.

*It still bothers me every time when shifts change. I don’t know who’s going to take care of me or how. There are agreements about someone coming to see me at the beginning of the shift, but it’s an emergency ward, so things often come up. And then it doesn’t always work out and I have a problem with that [female aged 29; general HIC]*.

Participants considered proper coordination between care providers, institutions and people from their social networks important for the recovery process, especially the coordination of the transition from inpatient to outpatient treatment. To ensure that a patient received well-organized, coordinated care, all the parties involved needed to know what was expected of them. This provided clarity and overview for everyone involved, preventing confusion, agitation, and overstimulation.

*I was discharged on the Monday and had an appointment with the outpatient team on the Tuesday. As I knew I’d have an appointment the day after discharge, the transition went well [female aged 25; ASD-HIC].*

Some participants reported that the process of recovery had been hindered by insufficient coordination.

*The problem is often transfer of information between caregivers, which doesn’t go smoothly, to put it mildly. It’s just that people are not always aware of either your crisis plan or other arrangements. Things like that [female aged 25 years; ASD-HIC]*.

### Process of recovery

The texts in italics in paragraphs below elaborate the process of recovery shown in the boxes in the central part of **Figure 1**. At the start their hospitalization, due mainly to the perceived severity of their problems and their often long and difficult treatment histories, most participants were pessimistic about the treatment provided and their chances of getting their lives back on track. But some had very high expectations. Since all or most of their earlier treatments and personal efforts had failed, they saw ASD-HIC treatment as a last resort – their only hope. As they also believed there would be no further options for treatment if this did not work out, and thus no future for them, they experienced considerable fear and pressure.

*If you feel that something is your last chance […], the pressure rises: you feel that it must succeed – and that you must do it well and that it must work. Everything has to work. Because if it doesn’t, there’s nothing left [female aged 25 years; ASD-HIC].*

For participants from ASD-HIC wards, the two preconditions referred to above – a good working alliance with care providers, and the clear and predictable organization of care – were met to a relatively high degree. As a result, they *experienced well organized care* and they *felt acknowledged and understood* as people with very individual characteristics, needs and preferences. This promoted personal feelings of trust and safety, which further increased their *trust in treatment and care providers*.

*So for me there’s more trust, more security if they can actually help me here and if they make a real effort to get to know me, to understand me. And then it all becomes a bit looser in your head and you have more space to take part in the treatment and learn a lot of things [female aged 25 years; ASD-HIC].*

Due to their increased confidence, participants reported that *they opened up to treatment and dared to express vulnerability.* This manifested itself in various ways: by being open and honest with themselves and those around them, by asking for help, and by discussing thoughts, functioning, problems or traumas.

*Yes, I could be very open and very honest with him. Of course, you don’t just talk to a complete stranger about your worst traumas – that’s quite a step to take [male aged 47; ASD-HIC].*

According to the participants, being vulnerable and open to treatment created *hope and new prospects* for treatment and their own future. These feelings were reinforced by positive experiences and the first successes in treatment, which contributed to more self-confidence, a positive self-image, and trust in positive treatment outcomes.

*If you notice that the simple things work, then you try the more difficult things, and if that works, then you get extra motivation. Then you experience success! [male aged 20; ASD-HIC].*

Because they had regained hope and prospects, participants were more confident about *actively collaborating with their care providers towards the treatment objectives*. Participants stated that they took new initiatives and tried things out by working toward these objectives step by step.

*Yes, really trying things out. What works for me and what doesn’t? Talking about it as well. And also seeing how others can help and support me in this [female aged 25; ASD-HIC].*

Participants said that active participation in treatment gave them *increased insight into themselves, their autism, and the people around them*. Growing confidence in care providers and treatment, and eventually in themselves, contributed significantly to a more goal-oriented attitude. This in turn contributed to greater understanding of their functioning, and greater awareness of own abilities and limitations. As a result, some participants realized that their goals and their expectations of themselves and of treatment had been too high.

*Sometimes it works, adjusting your goals. And also by simply having more insight and a little more understanding about what works differently with you than with others. These days, I adjust them, but I also know why I adjust them [female aged 25; ASD-HIC]*.

Most participants reported that increased understanding of their autism raised awareness of their disrupted or delayed information processing, their overstimulation and their specific thinking patterns.

*[In such cases], it was more like, I just don’t feel OK at all, and it just doesn’t work. But I didn’t know what caused it. Now I know it's because I don’t have the overview. And those panic attacks – they’re the result of over-stimulation. I’d never looked at it that way before [female aged 25; ASD-HIC]*.

Participants became more aware of their different information processing, which in turn *increased their scope for self-regulation*. They learned to take their personal characteristics into account, for example by allowing themselves sufficient time to process information, and to properly prepare and structure social situations and possible changes. They did so by trying to think in advance what was going to happen, by timely planning of appointments and activities, and by making a step-by-step plan.

*There are always sudden changes you can’t anticipate. And I still find it difficult to deal with them. But I do know that I must think, “OK, this has to sink in and be processed first. Come back in half an hour” [female aged 25; ASD-HIC].*

Participants reported that their self-regulation also increased, as they were better able to identify and regulate tension and overstimulation by setting limits to their activities and looking for distractions.

*I’ve learned more about my own limits and how to deal with them. I never really noticed them, but I do now. Not always, but now I do notice them more often, and I’m also more careful. I know that if I overstep them, I’ll always pay a price afterwards, as that’s when I always have to deal with over-stimulation [male aged 20; ASD-HIC].*

According to the participants, their self-regulation also increased because they had greater insight into and control over their thinking patterns. The blockages in their thinking had decreased, and they were more likely to discontinue negative or black-and-white thinking.

Participants also reported that, due their increased trust in their own judgements and choices, they were better able to take control of their own lives. In some areas of life, they also realized that they needed support for effective functioning, which is why, during treatment they developed skills such as discussing thoughts, emotions, functioning and problems, and asking for help and clarification when necessary.

*I now deal with the chaos in my head differently, especially by speaking out when it stops me from seeing clearly – asking others to help me make an overview of what’s going on in my head, what I can do about it at that moment, and what I have to do with it later [female aged 25; ASD-HIC].*

Ultimately, participants reported that they were *better able to accept their autism diagnosis and themselves*, and also their capabilities and limitations. This allowed them to adapt their lives accordingly, by adjusting goals and expectations based on their actual functioning and by accepting help from others.

*Part of it is the vulnerability and lack of social skills that get you into trouble. You must be very vigilant. You do need the help of those around you. You have to accept that you can’t do it alone. That’s your vulnerability [male aged 33; general HIC].*

The result was *improved autism management in daily life*, such as retaining a daily routine, carrying out education, sports, housework, and paid or voluntary work.

*I’ve picked up my studies again, and that’s the most important thing. For the rest, it’s all little things, like the contact with the sports club, as when I live at home again, I’ll start doing sports again. And I’m also looking for a job [male aged 20; ASD-HIC].*

Participants said it was crucial to their recovery that they had established a good daytime structure, with a good balance between rest and activity. In addition, they had noted a *decrease in their psychological and behavioral problems* throughout their recovery process, especially with regard to traumatic, depressive, anxiety or psychotic symptoms, suicidality, self-harm, aggression, alcohol and drug use, and the tendency to abscond.

*Yes, I just feel like doing things. Yes, I just want to pick up life again. To take life on again [female aged 25; ASD-HIC].*

By entering an upward spiral, with control over themselves and their surroundings gradually increasing, they had found themselves in an increasingly orderly world.

However, it was striking that this process of recovery was experienced mainly by participants from ASD-HIC and much less by those from general HIC, who indicated that the process of recovery had not been initiated, as the two essential conditions were usually missing: the establishment of a good working alliance, and the proper organization of care. Although some participants from general HIC had nonetheless had positive experiences with individual care providers who were able to establish a good working alliance, a more coherent treatment context with better conditions for recovery had been experienced by significantly more participants in specialized ASD-HIC.

## Discussion

The primary objective of our study was to investigate the process of recovery of adults on the autism spectrum during intensive inpatient treatment. Our model shows that participants experienced a world of confusion before intensive inpatient treatment. A world in which they had difficulties understanding themselves or others, and where they felt they were not well understood by the people around them. This significant mismatch between patients and others, both in their personal network and with care providers, resulted in a history of ineffective treatment.

Previous research confirms that persons on the autism spectrum often struggle to find services appropriate to their specific needs (11). The treatment currently provided in general mental health care facilities in the Netherlands seems insufficiently adapted to the specific needs of some persons on the autism spectrum, and thereby hampers the recovery process. Care providers in general mental health care seem to have less insight into persons on the autism spectrum, and less understanding of them. Eventually a reciprocal effect on the working alliance becomes apparent: both the care providers as well people on the autism spectrum can experienced challenges in understanding each other. Consequently, the interaction between them can be greatly affected. Due to the mismatch they experience between supply and demand, these people on the autism spectrum lose control over themselves and their surroundings.

The participants in our study felt like survivors, and many of them had developed behavioral problems prior to admission as a way of dealing with their problems and tension. In our experience, these dynamics in the development of behavioral problems often go unrecognized, and that this can lead to misinterpretation of a person’s behavior, and to stigmatization and misdiagnoses. Despite the difficulties they reported, participants were still determined to keep going as they tried to meet the standards they believed others expected of them by. For example, like many other non-autistic people in the Netherlands, people on the autism spectrum believe that when they reach the age of 18, they suddenly become adults, which “therefore” means that they are expected to be financially independent and to have a stable relationship. In adolescence, this naturally causes a lot of anxiety. Another feature is that people on the autism spectrum seemed to get stuck in a rigid mindset, seeing no way out without appropriate assistance. This is consistent with a failure to understand others and experiencing problems which require an overview over a specific situation, which is also described in cognitive explanatory models (27, 28).

When people on the autism spectrum demonstrate a rigid mindset, it is important to focus treatment on exploring with them the reasons for this rigidity, and on helping them to create more understanding and space to learn about new alternatives. By creating a safe and predictable context for engaging in new learning experiences, personalized treatment can support this. It is important for people on the autism spectrum to learn how and where they can explore, not just their own explanations for their experiences and behavior, but also alternative ones. The aim of treatment should therefore be not only to address present problems, but to teach persons the skills necessary to solving future problems themselves. To be able to do this, it is important that they discover how autism affects their thinking, functioning and interaction with those around them. This will enable them to better regulate their emotions and behaviors in daily life, and thereby gain more control over their own lives and reduce their dependence on care providers. No previous research has examined personal recovery in adults with ASD. However, various studies have been conducted into personal recovery within general mental health care. Interestingly, this previous (not autism-specific) research which holds that the aim of treatment is not about symptomatic recovery, but primarily about personal recovery (22, 24). Previous research also shows that it is important that seeing oneself as a person in recovery is a central part of the recovery process, associated with better wellbeing (18). Previous research also showed that recovery-oriented groups had benefits for attendee wellbeing and were valued for their role in fostering meaningful social connection (18). Our results did not indicate this, possibly because participants did not take part in specific recovery-oriented groups. During the analysis and when describing the results, we also continuously looked at ASD-specific elements in the recovery process. Previous research into recovery also indicated that recovery narratives are diverse and multidimensional (20). According to this previous research, there is no linearity and coherence between the different populations in mental health care and it is necessary to conduct research into recovery in various populations.

As discussed above, our model of recovery shows that there are two important preconditions for initiating the process of recovery: a good working alliance with care providers, and the clear, predictable organization of care. These preconditions must be met before therapy and goal-oriented treatment can start. It is essential that care providers have a proactive attitude towards both. Previous research has described the importance of the therapeutic relationship for treatment outcomes (29, 30). According to Hume (2022), the relationship does not receive enough attention in the context of working with adults with ASD (31). However, the authors mention that the therapeutic relationship is just as important for adults with ASD as for adults with other psychiatric problems. A change in perspective is needed to acknowledge that for adults with ASD relationships are important and that they should have the capacity for building relationships (31). Other studies also show that personalized care and a good therapeutic relationship for adults with ASD contribute to greater client satisfaction and better treatment results (32, 33).Our findings confirm that a good working alliance is particularly important for adults with ASD. As described above, one of the reasons people on the autism spectrum adopt a rigid stance in life is to help them create a foothold in an unpredictable world. But although sticking to one’s own rules and convictions may seem to reduce the confusion caused by the perceived unpredictability of others and limited understanding of one’s own lived experiences, it often clashes with the ideas of those without autism. To be willing to accept alternative perceptions on reality from someone else, a person on the autism spectrum needs a trustworthy relationship. This need is not just restricted to specialized autism treatment per se: the ability to develop such relationships should also be viewed as a basic competence of every care provider.

Given the importance of the quality of interpersonal contact for people on the autism spectrum, it is unsurprising that participants in the specialized ASD-HIC wards generally experienced better working alliances than those in the general HIC wards. ASD-HIC care providers are trained to combine these basic competencies with their autism expertise. To exchange and further develop their own autism expertise and expertise in their broader discipline, the care providers at the two ASD-HICs wards participating in this study collaborated closely and intensively on autism-specific treatment.

In addition to a good working alliance, a clear and predictable organization of care is important for recovery. This can also be achieved more easily in an ASD-HIC ward, as the content and organization of care are both more focused on autism than in a general HIC, where a wide range of psychiatric conditions must be dealt with, and where client turnover is also high. As our findings confirmed that predictability is a key element in the process of recovery. It is clear that the thorough intake phase before admission makes an important contribution to the conducive organization of care which participants experienced on the ASD-HIC wards. Unlike the acute, almost unconditional admission to a general HIC, this offered participants ample time to familiarize themselves with the unit. This may also explain why 80% of admissions to the general HIC are involuntary, against only 30% to the ASD-HIC.

Finally, the length of treatment may have contributed to the recovery process. At about nine months, the average stay at an ASD-HIC unit allowed for more time to establish a good working alliance and create understanding of how ASD impacts a person’s life than is possible in the few weeks or months in general HIC. As an ASD-HIC meets the conditions for recovery better than a general HIC, the many referrals and long waiting lists are unsurprising.

Although earlier literature (34) had led us to expect a distribution of three women to twelve men, ten of the fifteen participants in our study were women. However, a detailed examination of admission over recent years indicated that this gender distribution is representative of settings with autism-specific treatment. Most of our female participants reported that they had for many years been treated unsuccessfully for diagnoses other than ASD, which had added to their despair and their severe dysregulation. what may contribute to the long delays before starting with autism-specific treatment is that women on the autism spectrum tend to camouflage their social limitations with compensatory behavior, thereby obscuring the underlying autism (35, 36).

### Strengths and limitations

A strength of the study is that a topic list for the interviews was developed in advance and was based on discussions in focus groups representing three perspectives, experts by experience on the autism spectrum, family members, and caregivers. A second strength is that very little previous research has examined the recovery of persons in this target group. A third is that we studied recovery in various contexts by including participants from different settings in intensive inpatient treatment. A final strength is the cyclical nature of the data collection and analyses. Our attainment of data saturation showed the sample size to be sufficient.

A limitation is that the data presented are partial and concern only adults with need for admission High Intensive Care units due the severity of their autism symptoms, requiring very substantial support. Another limitation is that the generalizability of our results is restricted by our selection of two target group of adults on the autism spectrum: those in a general HIC, and those admitted in an ASD-HIC. The selected groups were very specific for the Dutch mental health care setting, which may not be easily translated to mental health care situations outside The Netherlands. However, the main conclusion is that adults with ASD emphasized that their need for being understood and accepted by caregivers - particularly with regard to their specific autism characteristics- is a prerequisite for treatment. Although this opinion was elicited in this specialist setting, we have no reason to assume that this need is different in other settings. Nevertheless, this finding needs to be replicated, and explored further. Another possible limitation is that the interviews were conducted two months after discharge, which may have been too soon to establish how participants functioned in daily life after intensive inpatient treatment. However, this interval was chosen deliberately in order to avoid recall bias.

### Clinical implications

Within the current mental health system in the Netherlands, treatment is based on standard protocols without either a comprehensive contextual assessment or an in-depth assessment of existing problems from the person’s perspective. Although this may be effective for the majority of the mental health population, this standard protocol-based approach is less appropriate for most people on the autism spectrum, as it assumes that there is agreement on the interpretations of existing problems and care needs, which is by no means the case for people on the autism spectrum.

Instead, it is important to explore together with the adults what the actual problems and related care needs are, paying explicit attention to their needs and lived experiences. In such assessments, people on the autism spectrum need to feel accepted and understood. Communication with them should therefore be tailored to their individual abilities and limitations – a reminder that comprehensive assessments and the establishment of a good therapeutic relationship lay the foundations for a proper recovery process.

Given the many referrals and long waiting lists, it is not possible to treat all eligible persons within a specialized ASD-HIC ward, as these specialized facilities are limited in number, and may not even exist in other countries. We therefore recommend that knowledge and competencies regarding proper assessments, effective communication and the overall facilitation of recovery of people on the autism spectrum are transferred to regular HIC units and other teams within regular mental health care. We also recommend that professionals at specialized ASD-HIC settings are made available for consultation in complex cases in regular mental health care. Such consultation and support from specialized ASD-HIC wards would also contribute to the transfer of specialist knowledge and competencies.

Before goal-oriented treatment can be provided, it is vital for people on the autism spectrum that a basis of trust is established. Such trust explains the advantages of prolonged treatment in ASD-HIC. In general HIC, persons are referred to another treatment setting after their crisis has stabilized, meaning that they once again have to get used to new care providers without first achieving the necessary basis in trust. We therefore recommend not only that care is provided using a care program or care pathway, but that a care provider is available who can follow each person on the autism spectrum through different settings and care pathways.

## Conclusions

There are two important preconditions for initiating the process of recovery of people on the autism spectrum during intensive inpatient treatment: a good working alliance with care providers, and a clear and predictable organization of care. These preconditions should be met before therapy and goal-oriented treatment can be carried out successfully. It is important for the recovery of people on the autism spectrum that they discover how autism affects their thinking, functioning and interaction with those around them, and that they learn in treatment how to apply these insights in their daily lives. By learning to regulate themselves in daily life, they will gain better control over their lives and become less dependent on care providers. Treatment should therefore focus on collaboratively exploring existing problems and related care needs, and on determining how these can be dealt with differently in daily life.

## Data availability statement

The raw data supporting the conclusions of this article will be made available by the authors, without undue reservation.

## Ethics statement

The studies involving humans were approved by Medical Ethics Review Committee at Amsterdam University Medical Center (UMC, location VUmc) (protocol no. 2017.120). The studies were conducted in accordance with the local legislation and institutional requirements. The participants provided their written informed consent to participate in this study.

## Author contributions

HB: Writing – original draft. BS: Writing – original draft, Writing – review & editing. EV: Writing – original draft, Writing – review & editing. AB: Writing – review & editing. BV: Writing – original draft, Writing – review & editing.
