# Supplementary material for: Recovery of adults with autism spectrum disorder during intensive inpatient treatment: a qualitative study
Source: Front Psychiatry. 2024 Jun 7;15:1383138. doi: 10.3389/fpsyt.2024.1383138 (PMC11190818; doi:10.3389/fpsyt.2024.1383138)
Supplement: Supplementary file 1 [file DataSheet_1.docx]

**Appendix**

**Interview protocol and topic list**

*3 Main questions:*

1. You were admitted to the ASD-HIC or general HIC ward some time ago. Can you tell us how you’re doing now?

2. Can you go back in your mind to a period before you were admitted, i.e., the moment you were in the crisis that made your admission necessary? Can you describe how you were doing just before you were admitted?

3. Recovery is a very gradual process that’s different for each person. Can you tell us how the recovery process went for you, starting from the beginning of your stay on the ward and ending today? Shall we start at the beginning? When did you first feel you were doing a little better? How did you notice it? What had changed? How did it come about? What had helped make this recovery start? Were there also things that had a negative effect on it?

*Topics during the interviews*

Participants’ perspectives:

- Which possibilities participants thought were open to them and which successes they had had
- Their overall perspectives for the future
- The extent to which they participated in society

Participants’ self-perception and coping (regarding increases in self-insight and self-regulation):

- Self-acceptance
- Extent to which they could manage their own lives
- Extent to which treatment had increased their self-regulation skills
- Extent to which they had gained and were still gaining control over their lives
- Degree of self-insight and awareness that therapy had brought
- Degree of self-confidence that therapy had brought
- Current control over symptoms
- Functional disabilities
- How the treatment had affected their motivation for treatment

Other personal aspects:

- Intelligence
- Psychiatric comorbidity
- Health problems including sleep problems

Coordination and interaction with the people around them:

- How much support they got from those around them
- Information about family/friends/day care/living situation/finances
- The extent to which those around them had been involved in their treatment or its coordination

Participants’ perceptions of the autism expertise of their care providers

Participants’ perceptions of the working alliance with their care providers:

- Whether their care providers had sought real contact and connection
- Whether they had offered recognition and trust
- Whether they had approached participants as full human beings
- Whether they had approached participants as equals
- Whether they had been available for contact
- Whether they had had full attention for the whole person and their context

Organization of care:

- The extent to which specialized ASD care had been available
- The extent of continuity and coordination
- Whether a safety net had been provided by care providers and relatives, and how great its quality had been

The effectiveness of treatment with regard to:

- Contact with one’s own body
- Physical release
- Writing as therapy or as a way of seeking or establishing contact (e.g., through a life story)
- Therapy through action rather than through talk
